# Supplementary material for: Hippocampal and Cortical Pyramidal Neurons Swell in Parallel with Astrocytes during Acute Hypoosmolar Stress
Source: Front Cell Neurosci. 2017 Sep 20;11:275. doi: 10.3389/fncel.2017.00275 (PMC5611379; doi:10.3389/fncel.2017.00275)
Supplement: Supplementary file 1 [file Presentation_1.zip › Supplementary Movie S1/Supplementary Movie S1 legend.docx]

**Supplementary Movie S1**. **Neuron and astrocyte swelling during repeated applications of 17% and 40% hACSF.** Representative CA1 pyramidal neurons (top) and stratum radiatum astrocytes (bottom), loaded with fluorescent dye via patch clamp and imaged over three successive applications of Mg^2+^-free hACSF + TTX + NBQX (compare to graphs in Figure 2). Astrocytes and neurons swelled to similar degrees and in a dose-dependent manner when exposed to 17% and 40% hACSF. Note characteristic “dimming” of the fluorescent indicator as water enters the cells during exposure to hACSF, as well as the recovery of cell volume to near baseline levels during intervening wash periods in normosmolar ACSF. Please refer to results for Figure 2 for more details.
